# Coix Seed-Based Milk Fermented With *Limosilactobacillus reuteri* Improves Lipid Metabolism and Gut Microbiota in Mice Fed With a High-Fat Diet

**DOI:** 10.3389/fnut.2022.921255

**Published:** 2022-07-12

**Authors:** Zhoujie Yang, Xiaoli Zhu, Anyan Wen, Jingqi Ran, Likang Qin, Yi Zhu

**Affiliations:** ^1^Key Laboratory of Plant Resource Conservation and Germplasm Innovation in Mountainous Region (Ministry of Education), College of Life Sciences/Institute of Agro-Bioengineering, Guizhou University, Guiyang, China; ^2^School of Liquor and Food Engineering, Guizhou University, Guiyang, China; ^3^Plant Protection and Plant Quarantine Station of Guizhou Province, Guiyang, China

**Keywords:** coix seed milk, *Limosilactobacillus reuteri*, HFD, hyperlipidemia, intestinal microbiota

## Abstract

The aim of this study was to investigate the effects of coix seed-based milk (CSM) fermented with *Limosilactobacillus reuteri* (*L. reuteri*) on dyslipidemia and the composition of the intestinal microbiota in high fat diet (HFD)-fed mice. Changes in the body weight, serum lipid levels, activities of hepatic oxidative stress factors, expression of lipid-related genes, and composition of the intestinal microbiota of HFD-fed mice after supplementation with CSM were determined. The results showed that intake of CSM reduced the body weight gain as well as serum total cholesterol (TC), triglyceride (TG), and low-density lipoprotein cholesterol (LDL-C) levels, and increased the high-density lipoprotein cholesterol (HDL-C) levels in the mice. Meanwhile, supplementation with CSM could relieve liver oxidative stress, down-regulate the expression of genes related to lipid synthesis, and prevent liver fat accumulation in mice fed with HFD. The 16S rRNA sequencing of the intestinal microbiota showed that CSM regulated the gut microbiota community structure at different taxonomic levels, and reversed gut dysbiosis induced by HFD. The relative abundance of *Muribaculaceae, Lachnospiraceae, Dubosiella* and *Akkermansia* which are negatively correlated with blood lipid levels were significantly increased by the intervention of CSM, while the relative abundance of *Desulfovibrionaceae, Ruminococca-ceae_UCG-014, Psychrobacter*, and *Staphylococcus* which have positive correlation with blood lipid levels were significantly decreased. These results indicated that CSM might serve as a novel and promising dietary supplement for ameliorating hyperlipidemia and intestinal microbiota disorders caused by HFDs.

## Introduction

Hyperlipidemia is a metabolic syndrome caused by abnormal lipid metabolism or lipid transportation and is mainly characterized by elevated serum total cholesterol (TC), triglyceride (TG), and low-density lipoprotein cholesterol (LDL-C) levels (1). The incidence of hyperlipidemia has increased substantially as a result of changes in living standards and lifestyles associated with reduced exercise and dietary transformations; as such, hyperlipidemia has emerged into a global disease that endangers public health. Epidemiological studies have shown that hyperlipidemia increases the risk of developing several other chronic diseases, including obesity (2), diabetes (3), and cardiovascular disease (4). Although some drugs have been developed to treat hyperlipidemia, they are not a viable, long-term solution because these drugs not only might engender chronic side effects but also create a heavy economic burden on patients (5). Instead, dietary intervention might may be the most effective strategy for preventing and treating of hyperlipidemia, which, fortunately, has been corroborated by several studies (6–8).

In recent years, a significant amount of interest has been stimulated around the anti-hyperlipidemia effect of certain functional foods and probiotics (9, 10). The active components of functional foods, such as polyphenols, polysaccharides, lipids, proteins, and saponins, have been shown to decrease blood lipid levels (11). Functional cereals, as one of the functional foods, play an indispensable role in regulating blood lipid levels. Previous study found that supplementing quinoa polysaccharides to the high-fat diets (HFD) of obese rats resulted in the attenuation of weight gain and decreased in serum TG, TC, and LDL-C levels (12). Xia et al. found that coix seed polysaccharides not only increased serum insulin and HDL-C levels but also decreased the levels of TC, TG, and LDL-C; activated the IGF1/PI3K/AKT signaling pathway and generated hypoglycemic effects (13). Ji et al. showed that the dietary intake of a mixture of coarse cereals led to a decrease in fat accumulation and systemic inflammation as well as a downregulation in the expression of hepatic lipogenic genes (14). These studies corroborated that increasing the intake of functional cereals might serve as a practical dietary intervention strategy for curbing hyperlipidemia.

In addition to functional cereals, the anti-hyperlipidemia efficacies of probiotics have been widely studied. Several studies indicated that probiotics might induce anti-hyperlipidemia effects by promoting fatty acid oxidation, reducing lipid-related gene expression, and regulating intestinal microbiota (15, 16). *Bifidobacterium longum* has been shown to reduce serum TG, TC, and LDL-C levels and enhance the activity of superoxide dismutase (SOD) in the liver of HFD-fed mice (17). Clinical experiments indicated that *L. reuteri* reduced the intestinal absorption of free fatty acids and promoted the fecal excretion of free fatty acids (18). Growing evidence also suggests that probiotics have the potential to display hypolipidemic effects by regulating intestinal microbiota (19, 20).

The development of functional foods containing probiotics and functional cereals has become a research hotspot in food science. In particular, attention is being placed on utilizing these functional foods to prevent and treat many chronic diseases (21, 22). Wang et al. found that *Lactobacillus*-fermented cereal pastes significantly reduced serum and liver cholesterol concentrations, raised the antioxidant capacity of the serum, and balanced microbial populations in hamsters (23). One recent study indicated that kefir fermented soy milk exhibited promising beneficial effects on obesity, hyperlipidemia, and hyperglycemia and prevented obesity-related toxicity in the liver and kidneys on HFD fed rats (24). Wang et al. research found that adlay-based non-dairy milk fermented with *Lactobacillus plantarum* or *Lactobacillus paracasei* had the effect of lowering serum cholesterol and defensed of hyperlipidemia-induced oxidative stress (25). However, to date, no reports regarding the effects of coix seed milk (CSM)-fermented with *L. reuteri* to ameliorate blood lipids and intestinal microbiota have been reported.

In this study, the effects of CSM on diet-induced hyperlipidemia in mice were investigated by evaluating the changes in the serum lipid profile, fat accumulation in the liver, and expression of certain genes related to lipid metabolism, and the possible mechanism of CSM in regulating blood lipids was explored. Finally, the effects of CSM on the composition of the gut microbiota were assessed by 16S rRNA sequencing of the cecal contents of the mice.

## Materials and Methods

### Materials and Reagents

Coix seeds were obtained from Guizhou Renxin Agriculture Development Co., Ltd. (Guizhou, China). *L. reuteri* BNCC186563 was used as the probiotic organism and purchased from BeNa Culture Collection (Suzhou, China). The strain was sub-cultured three times for 24 h at 37°C in Man Rogosa Sharp broth (MRS; Shanghai Bio-Way Technology Co., Ltd., Shanghai, China) before each experiment. The starter culture was cultured and inoculated for 16 h at 37°C in MRS broth, then centrifuged at 3000 rpm for 5 min, washed with 0.9% saline, and diluted with 0.9% saline to obtain a solution with a concentration between 10^7^ and 10^8^ CFU/mL. Ultrapure water was used to prepare all aqueous solutions. All other reagents were of analytical or chromatography grade and purchased from Sinopharm Chemical Reageant Co., Ltd. (Suzhou, China).

### Preparation and Fermentation of Coix Seed Milk

Coix seeds (50.0 g) were soaked in distilled water at a ratio of 1:10 (seed to water) for 3 h at room temperature. After soaking, the cereals were ground in an electric grinder and filtered through 120 mesh cloth. The filtered suspension was steamed at 105°C for 10 min and cooled to 37°C, inoculated 5% probiotic cultures (10^7^ CFU/mL of bacteria) and fermented for 24 h at 37°C.

### Animal Groupings and Feeding

Specific pathogen-free (SPF) male Kunming mice (20.0 ± 2.0 g, SCXK (jing) 2019-0010) were obtained from Spaefer (Beijing) Biotechnology Co., Ltd. (Beijing, China). The mice were kept in a room with a stable environment (temperature: 25°C, humidity: 60%, conventional 12-h light/dark cycle) and had free access to water and food. After 1 week of adaption, the mice were randomly divided into five groups (*n* = 10 per group): NC group [mice were fed normal diet (Purchased from Beijing HFK Bioscience Co., Ltd)], HFD group (mice were fed HFD), HFDF group (mice were fed HFD and fermented coix seed milk with *L. reuteri*), HFDUF group (mice were fed HFD and unfermented coix seed milk), HFDLr group [mice were fed HFD and *L. reuteri* (the viable count was 9 Log10 CFU/mL)] (4). The normal control (NC) group of mice were fed normal diet during the entire experiment, while the experimental group of mice were fed a HFD (1% cholesterol, 10% lard, 10% egg yolk powder, and 0.2% cholate were added into normal diet). From week 5 to week 8, different experimental groups were given different samples for intervention (10 mL/kg body weight per day). The body weight of the mice was measured weekly. All mice were sacrificed by asphyxiation by diethyl ether inhalation at the end of the eighth week. Blood was collected from the orbital vein and left to stand for 2 h before centrifugation at 3,000 g for 10 min, after which the serum was collected and stored at −80°C for analysis. The livers and spleens were excised, weighed, washed with sterile saline, and stored at −80°C. The values of the liver indices and spleen indices were calculated as the ratio of mass of the liver and spleen to the total body weight of the mice [Formula: organ index = organ weight (g)/body weight (g)]. The cecal contents were also collected and stored at −80°C for 16S rRNA sequencing. All experimental procedures were followed according to the laboratory animal welfare standards and approved by the subcommittee of experimental animal ethics at Guizhou University (No. EAE-GZU-2020–P011).

### Biochemical Analysis of Serum Lipids

Serum levels of TC, TG, HDL-C, and LDL-C were measured using biochemical kits [obtained from Nanjing Jiancheng Bioengineering Institute (Nanjing, Jiangsu, China)].

### Liver Histological Analysis

Each of the fresh livers (1 × 1 × 1 cm in size) was fixed in 4% paraformaldehyde for 24 h, embedded in paraffin after gradient dehydration, and cut into sections 4 μm thick. The sections were stained with hematoxylin and eosin (H&E) and Oil Red O (Wuhan Servicebio Technology Co., Ltd., Wuhan, China) to observe histopathological changes and the degree of fat accumulation under an optical microscope (Ningbo Sunny Intruments Co., Ltd., Zhejiang, China).

### Oxidative Stress Analysis of the Liver

Livers (0.1 g) were homogenized in 0.9 mL of saline solution in an ice-water bath and centrifuged (3,000 rpm for 10 min), after that, the supernatant was taken for assaying various indicators of oxidative stress. The concentrations of proteins in the liver homogenates were determined using Bradford assays (Beijing Solarbio Science Technology Co., Ltd., Beijing, China). The MDA content in the liver homogenates, as well as the activities of the antioxidant enzymes SOD, GSH-Px, and CAT, were evaluated using biochemical kits (Nanjing Jiancheng Bioengineering Institute, Nanjing, Jiangsu, China) according to the manufacturer's instructions.

### Quantitative Real-Time Polymerase Chain Reaction (qPCR) Analysis

The total RNA in the liver was isolated using the RNAprep pure tissue kit (Tiangen biotech Co., Ltd., Beijing, China) according to the manutacturer's instructions. The RNA purity was determined by measuring OD_260_/OD_280_ ratio (between 1.8 and 2.1). Reverse transcription was performed using the FastKing gDNA Dispelling RT SuperMix (Tiangen biotech Co., Ltd., Beijing, China) using the following conditions: 42°C for 15 min followed by 95°C for 3 min. Quantitative real-time PCR (qPCR) was performed using the Talent qPCR PreMix (SYBR Green) (Tiangen Biotech Co., Ltd., Beijing, China) under the following conditions: 95°C for 5 min followed by 40 cycles at 95°C for 5 s, 50–60°C for 10 s, and 72°C for 15 s. The CT values for the target genes and β-actin gene (as a housekeeping gene) were calculated by the real-time PCR instrument (Bio-Rad, USA), and the relative gene expression of four genes involved in lipid metabolism, including acetyl-CoA carboxylase (ACC), fatty acid synthase (FAS), HMG-CoA reductase (HMGCR) and cholesterol 7 alpha-hydroxylase (CYP7A1) was calculated using the comparative method 2^−ΔΔ^CT (26). The primers sequences used for qRT-PCR in this study were obtained from ThermoFisher (Beijing, China) and shown in Table 1.

**Table 1:** Primer sequences used for qPCR.

**Gene**	**Forward primer (5^**′**^-3^**′**^)**	**Reverse primer (5^**′**^-3^**′**^)**
β-actin	GGGAAATCGTGCGTGAC	AGGCTGGAAAAGAGCCT
ACC	TTGGACAACGCCTTCAC	GCAGCCCATTACTTCATCA
FAS	CCAGTCGTGAAACCATACC	TCTTGCCCTCCTTGATGT
HMGCR	GACGCTCTTGTGGAATGC	ATTGGACGACCCTCACG
CYP7A1	GGGATGTATGCCTTCTGCT	AGTGCCGGAAATACTTGGT

### Gut Microbiota Analysis

The cecal contents were removed by scraping under aseptic conditions for intestinal microbiological analysis. DNA was extracted from the cecal contents using the Magnetic Soil and Stool DNA Kit (Tiangen biotech Co., Ltd., Beijing, China) according to the manutacturer's instructions. The full-length of the bacterial 16S rRNA gene was amplified using the following primer pairs: 27F (5′-AGRGTTTGATYNTGGCTCAG-3′) and 1492R (5′-TASGGHTACCTTGTTASGACTT-3′) (27). Thermal cycling consisted of the following condition: 95°C for 2 min; 25 cycles of 98°C for 10 s, 55°C for 30 s, 72°C for 1 min 30 s; and a final extension period at 72°C for 7 min. After amplification, the PCR products were subjected to agarose gel electrophoresis (1.8% agarose) to test for integrity. High-throughput pyrosequencing of the PCR products were performed on the pacBio sequel I platform Technologies Co., Ltd. (Beijing, China). The raw paired-end reads from the original DNA fragments were merged by FLASH (28), and the sequences were assigned to each sample according to a unique barcode. Possible chimeric sequences were identified using the UCHIME algorithm. The sequences were clustered into operational taxonomic units (OTUs) using the QIIME UCLUST module based on a 97% sequence similarity. Taxonomy was assigned to all OTUs by searching against the Silva databases using the RDP classifier within QIIME. For alpha diversity analysis, Chao 1 and Simpson indices were assessed to observe the community diversity and community richness. Beta diversity was assessed by non-metric multidimensional scaling (NMDS) within QIIME to investigate structural variations of the microbial communities between the sample groups. The microbial abundance of bacteria at various classification levels, including phylum, class, order, family, genus, and species, was analyzed by multivariate statistical analysis, the results from which were compiled to create a relative abundance histogram of the bacterial communities that was drawn using the R program. Spearman correlation analysis was used to evaluate the correlation coefficient between intestinal microflora and lipid level parameters.

### Statistical Analysis

All results were reported as the mean ± standard deviation (SD). Statistical analysis was performed by the SPSS statistics 19.0 software package (IBM, USA). The statistical differences between groups were analyzed by analysis of variance (ANOVA) followed by Duncan's tests. A value of *p* < 0.05 was considered statistically significant.

## Results

### CSM Intervention Ameliorated the Weight Parameters and Viscera Index

**Figure 1:**
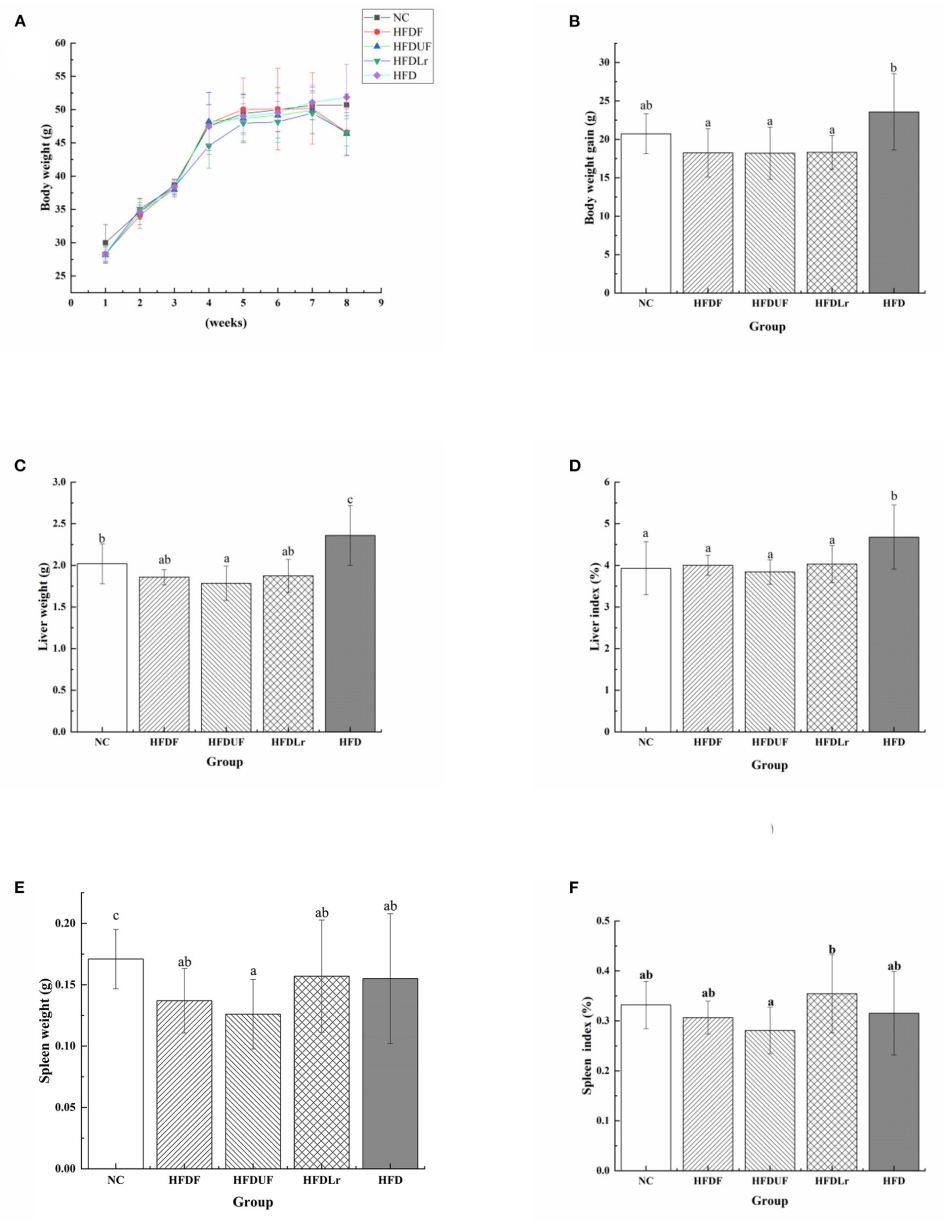
The effects of CSM on body weight and viscera index of HFD-fed mice. **(A)** Body weight, **(B)** body weight gain during the experiment, **(C)** liver weight, **(D)** liver index, **(E)** spleen weight, **(F)** spleen index. All data are expressed as the mean ± SD (n = 10 mice/ group). The statistical differences between groups were analyzed using ANOVA. A *p* value < 0.05 was considered statistically significant. The superscript letters indicate significant differences between groups.

Consuming a HFD is a significant cause of rapid weight gain. Therefore, the effects of supplementation of the HFD with CSM on the body weight as well as liver and spleen indices of the HFD-fed mice were evaluated. As shown in Figure 1, before dietary intervention (from week 1 to 4), consumption of a HFD diet resulted in a rapid increase in body weight in each group. However, after dietary intervention (from week 5 to 8), the increase in body weight of the experimental groups occurred at a much slower rate compared to before intervention (Figure 1A). Notably, the weight of the mice decreased in the experimental group between weeks 7 and 8. Compared to the HFD group, CSM intervention led to a significant reduction in the body weight of the mice (*p* < 0.05). Similar results were observed with unfermented coix seed milk (HFDUF) and *L. reuteri* (HFDLr) (Figure 1B). There was no statistically significant changes in body weight of the mice in the HFDF, HFDUF, and HFDLr groups (Figure 1B). In addition, the masses of the liver and liver index of the mice in the HFD group were significantly higher than in the other groups (Figures 1C,D) (*p* < 0.05). Compared with the NC group, there was no significant difference in liver weight and liver index between the HFDF, HFDUF and HFDLr groups. Although the spleen weight and spleen index of mice given CSM and unfermented coix seed milk were not statistically different from those in the HFD group (*p* > 0.05), they were lower than those in the HFD group (Figures 1E,F). Compared with HFD group, there were no significant changes in spleen and spleen index of mice fed with *L. reuteri*.

### Effects of CSM on Serum Lipid Levels

**Figure 2:**
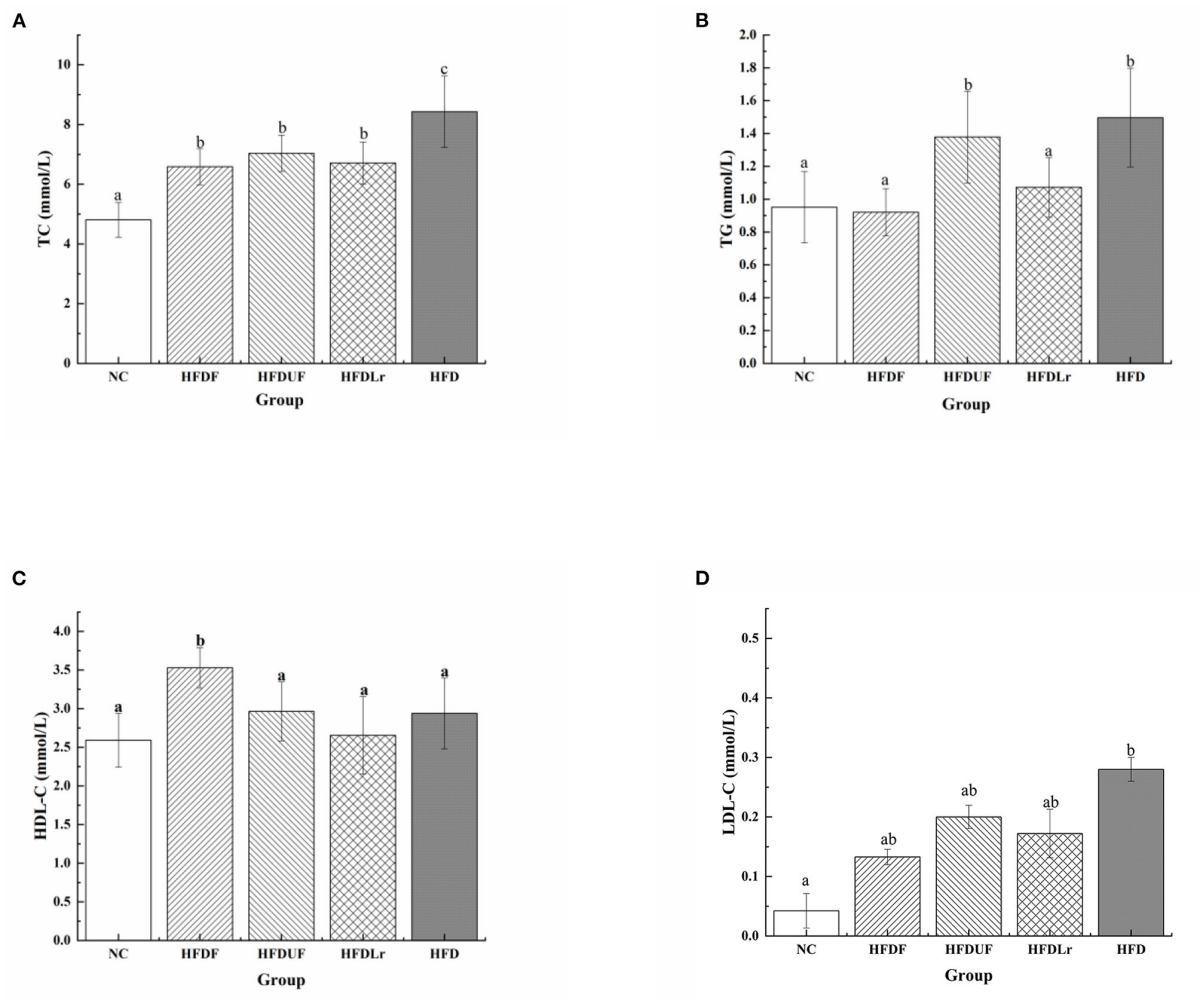
The effects of CSM on serum lipid levels. **(A)** Total cholesterol (TC), **(B)** triglyceride (TG), **(C)** high-density lipoprotein cholesterol (HDL-C), and **(D)** low-density lipoprotein cholesterol (LDL-C). All data are expressed as the mean ± SD (*n* = 10 mice/group). The statistical differences between groups were analyzed using ANOVA. A *p* value < 0.05 was considered statistically significant. The superscript letters indicate significant differences between groups.

To evaluate the effects of CSM on the serum concentrations of lipids, the changes in the serum concentrations of TC, TG, HDL-C, and LDL-C were measured in the HFD-fed mice after CSM supplementation. Compared with the NC group, the HFD engendered significant increases in the serum TC and TG levels in the mice (Figures 2A,B). However, after 4 weeks of dietary intervention with CSM, the serum concentrations of TC, TG, and LDL-C in the mice were significantly reduced compared with the HFD group (*p* < 0.05). Moreover, CSM significantly increased the level of HDL-C in serum of HFD fed mice (*p* < 0.05) (Figure 2C). However, there were no significant differences in the TC and LDL-C levels among the HFDF, HFDUF, and HFDLr groups of mice (*p* > 0.05). Compared with HFD group, the serum TC level of the HFDUF group was significantly decreased (*p* < 0.05) (Figure 2A), but TG level was not significantly different (Figure 2B). In addition, there was no significant difference in HDL-C and LDL-C between HFDUF group and HFD group (Figures 2C,D). Nevertheless, the HDL-C level in HFDUF group was still higher than that in HFD group, and the LDL-C level was lower than that in HFD group. It was suggested that the intake of unfermented coix seed milk had a certain regulatory effect on serum lipid level in high fat diet. The serum TC and TG levels in mice treated with *L. reuteri* were significantly lower than those in the HFD group (*p* < 0.05). Notably, *L. reuteri* did not significantly increased HDL-C levels compared to HFD group, but decreased LDL-C levels. Taken together, intake of CSM, unfermented coix seed milk and *L. reuteri* could regulate the serum lipid level of high-fat diet mice. In contrast, intake of CSM had a more significant regulatory effect on serum lipid levels.

### Effects of CSM on MDA Levels and SOD, CAT, and GSH-PX Activities in the Liver

**Figure 3:**
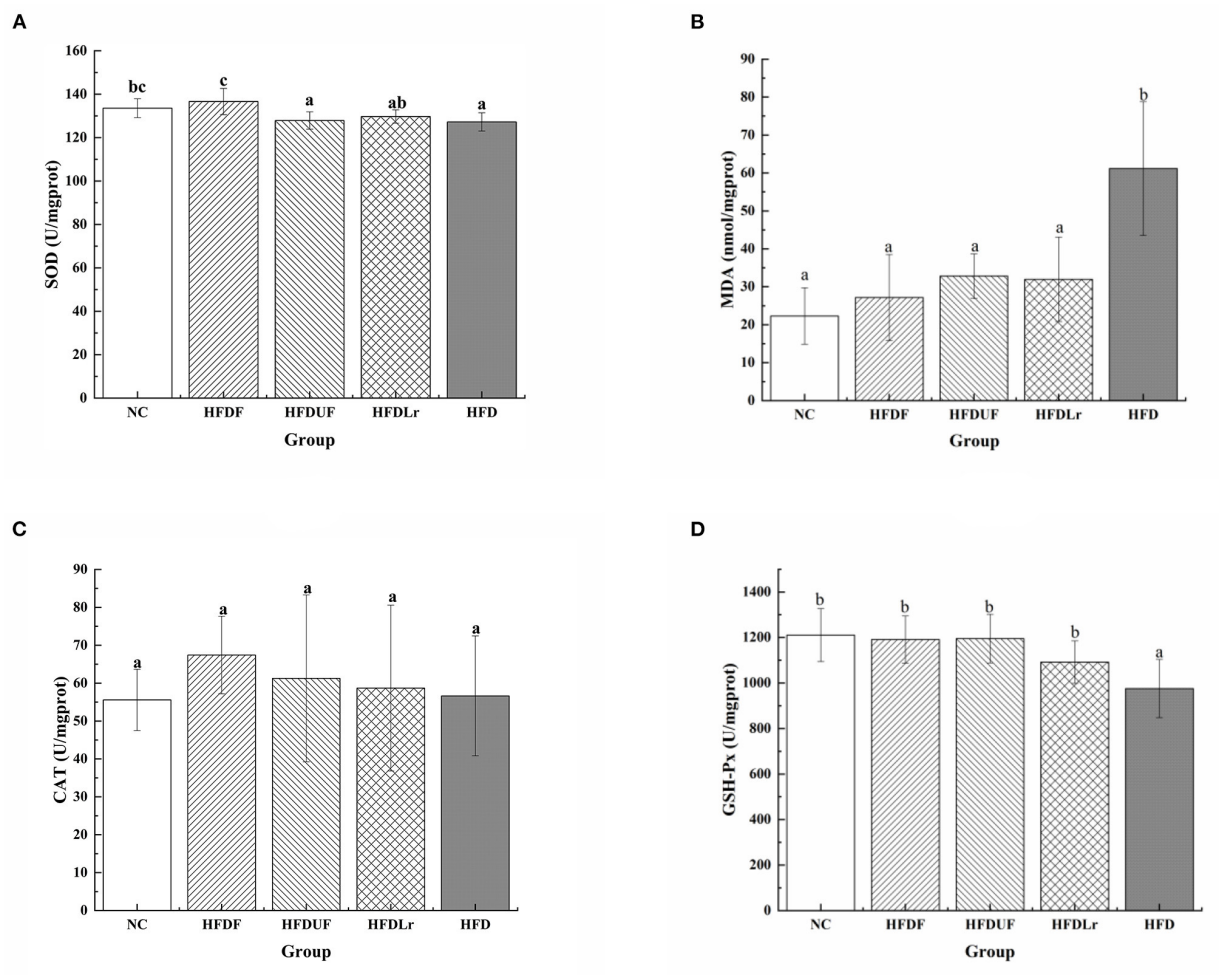
The effects of CSM on the activities of selected antioxidant enzymes in the liver and expression of malondialdehyde (MDA). **(A)** Superoxide dismutase (SOD), **(B)** MDA, **(C)** catalase (CAT), and **(D)** glutathione peroxidase (GSH-Px). All data are expressed as the mean ± SD (*n* = 10 mice/ group). The statistical differences between groups were analyzed using ANOVA. A *p* value < 0.05 was considered statistically significant. The superscript letters indicate significant differences between groups.

As shown in Figure 3, compared with the HFD group, CSM significantly increased the activity of SOD and decreased the production of MDA in the liver of the HFD-fed mice (*p* < 0.05) (Figures 3A,B). The activity of CAT was increased in HFDF group (Figure 3C), but there were no significant differences in the CAT activity between the HFDF group and the other groups (*p* > 0.05). In addition, the activity of GSH-Px was significantly enhanced in the HFDF group compared to the HFD group (*p* < 0.05) (Figure 3D). Compared with the HFD group, supplementation with unfermented coix seed milk (HFDUF group) and *L. reuteri* (HFDLr group) did not significantly increase the activity of SOD (Figure 3A), but significantly decreased the production of MDA (*p* < 0.05) (Figure 3B). In addition, the CAT activity of HFDUF group and HFDLr group was not statistically different from that of HFD group, but the activity of GSH-Px was significantly higher than that of HFD group (*p* < 0.05) (Figure 3D). In general, although CSM, unfermented coix seed milk, and *L. reuteri* all increased the activity of antioxidant enzymes in the liver of mice fed a high-fat diet, CSM had the most significant effect. These results suggested that CSM increased antioxidant enzyme activities, thereby reducing oxidative stress in the liver of HFD-fed mice.

### Effect of CSM on the Liver Histopathology

**Figure 4:**
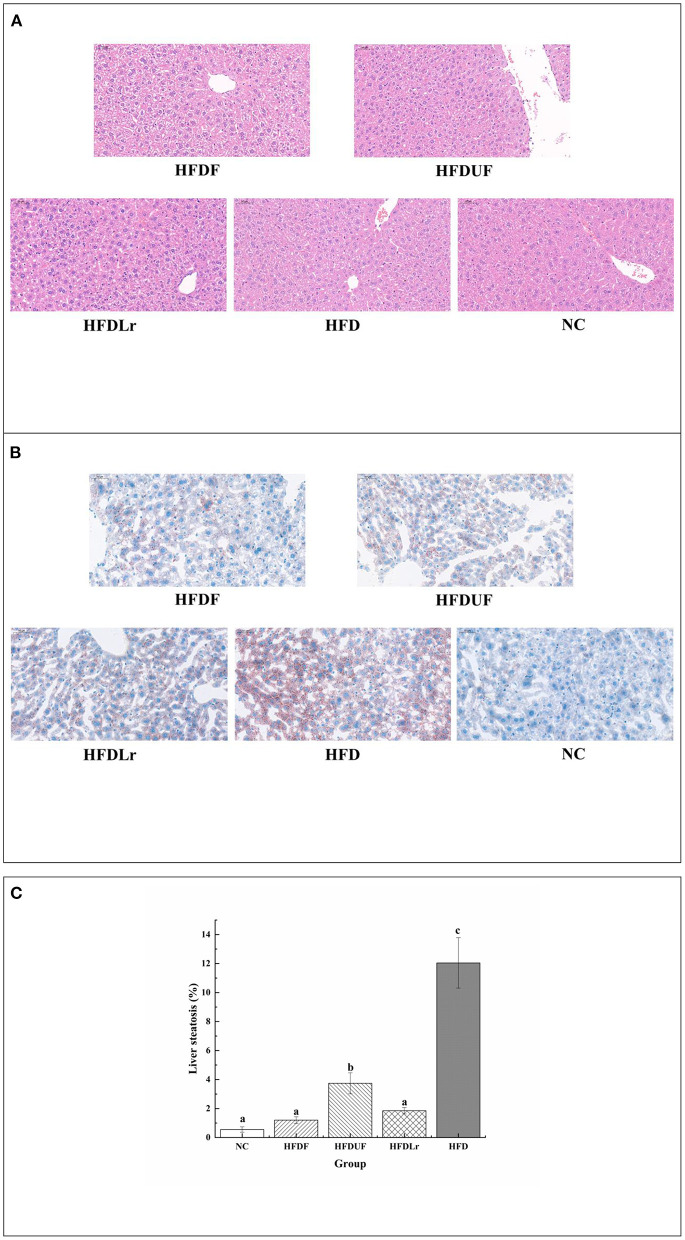
The effect of CSM on liver histopathology. **(A)** Liver morphology was assessed by H&E staining (200× magnification). **(B)** Liver steatosis was assessed by oil-red O staining (200× magnification). **(C)** Liver steatosis ratio stained with oil-red O staining. Scale bars: 50 μm. Data are expressed as the mean ± SD (*n* =3 mice/ group). The statistical differences between groups were analyzed using ANOVA. A *p* value < 0.05 was considered statistically significant. The superscript letters indicate significant differences between groups.

Figure 4 displays the microphotographs of the liver tissue from different groups after H&E staining and oil red O staining. As indicated by the images, there were no obvious pathological changes or steatosis in the liver of the NC mice group. In contrast, the liver tissue of the mice in the HFD group showed noticeable liver injuries, including enlargements of hepatocytes as well as vesicular steatosis, suggesting that HFD consumption promoted fat deposition in the liver (Figure 4A). Compared with the HFD group, the liver tissue of the HFDF group mice had a well-organized structure, and the number of fat vacuoles was significantly lower, indicating that hepatic steatosis and lipid vacuolization were obviously alleviated by CSM supplementation (Figure 4B). Meanwhile, the HFDUF and HFDLr groups of mice exhibited reduced hepatic fat lesions than the HFD group. Compared with HFDUF group and HFDLr group, the degree of liver steatosis in HFDF group was lower (Figure 4B). The rate of liver steatosis was calculated based on oil red O staining (Figure 4C). The liver steatosis rate of NC group (0.55 % ±0.19) was the lowest, while that of HFD group (12.04% ± 1.74) was the highest. The liver steatosis rates of mice in HFDF group and HFDLr group were 1.19% ± 0.23 and 1.85% ± 0.22, respectively, which were significantly lower than those in HFDUF group (3.74% ± 0.73). Based on these experimental results, CSM, unfermented coix seed milk, and *L. reuteri* all effectively inhibited fat accumulation in the liver, while CSM had the most obvious inhibitory effect on liver steatosis in HFD fed mice, indicating that CSM exerted a certain protective effect on the liver.

### Effects of CSM on the Expression of Lipid-Related mRNA Genes in the Liver

**Figure 5:**
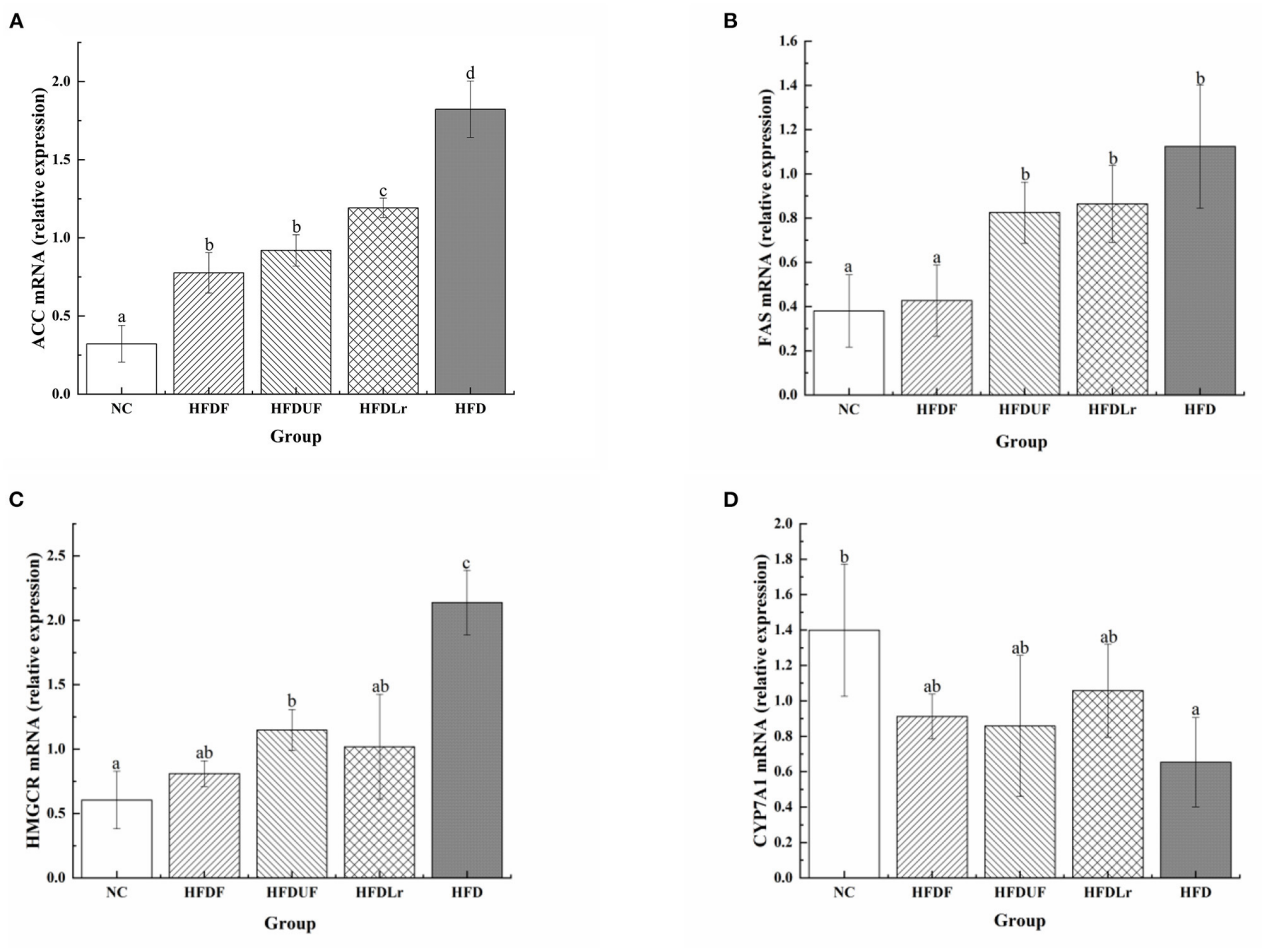
The effects of CSM on the mRNA expression of lipid metabolism-related genes in mice livers. **(A)** acetyl-CoA carboxylase (ACC), **(B)** fatty acid synthase (FAS), **(C)** HMG-CoA reductase (HMGCR), **(D)** cholesterol 7 alpha-hydroxylase (CYP7A1). The mRNA levels were determined using real-time quantitative PCR (qPCR) and normalized to the mRNA expression levels of β-actin. All data are expressed as the mean ± SD (*n* = 10 mice/ group). The statistical differences between groups were analyzed using ANOVA. A *p*-value < 0.05 was considered statistically significant. The superscript letters indicate significant differences between groups.

To evaluate the hypolipidemic effect of CSM, the changes in the expression of lipid metabolism-related genes were determined by qPCR, including ACC, FAS, HMGCR, and CYP7A1. As shown in Figure 5A, the mRNA levels of ACC were significantly lower in the HFDF group than in the HFD group (*p* < 0.05), while there were no significant differences in the ACC mRNA levels between the HFDF and HFDUF groups. The ACC mRNA levels in the HFDLr group were also significantly lower than in the HFD group (*p* < 0.05). In addition, the mRNA expression of FAS genes in the HFDF group was significantly lower than in the HFD group but comparable to the NC group (Figure 5B). Notably, there were no significant differences in the mRNA expression levels of FAS genes between the HFDUF, HFDLr, and HFD groups. In addition, the mRNA expression of HMGCR was significantly reduced in the HFDF group compared to the HFD group (*p* < 0.05) (Figure 5C). In contrast, the mRNA expression of CYP7A1 was higher in the HFDF group compared to the HFD group (Figure 5D). These results indicated that CSM reduced the expression of genes related to liver fat synthesis, corroborating the hypolipidemic effect previously hypothesized.

### Effects of CSM on Intestinal Microbiota

**Figure 6:**
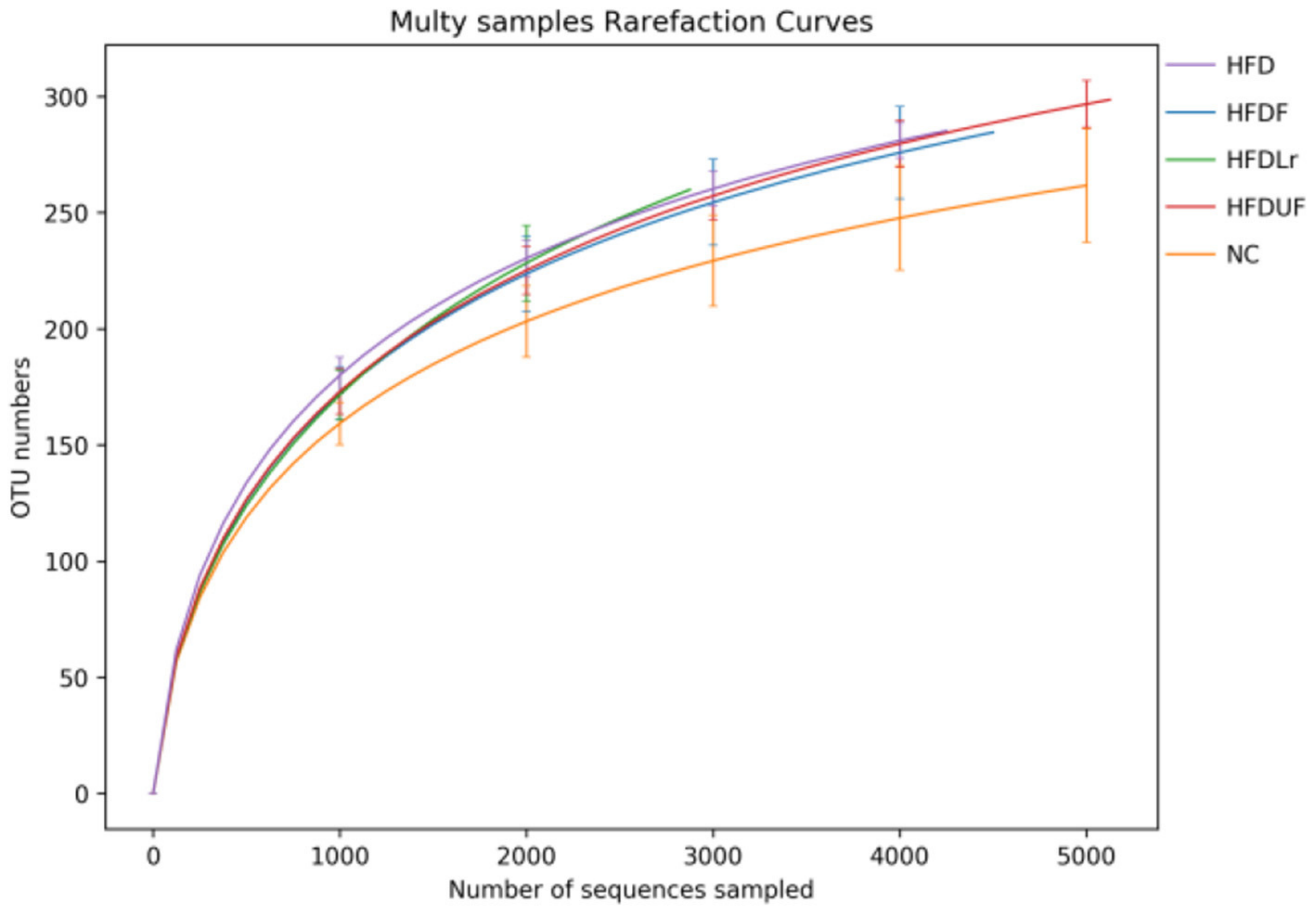
Rarefaction curves derived from the 16S rRNA sequencing of the microbial species in each sample group.

**Figure 7:**
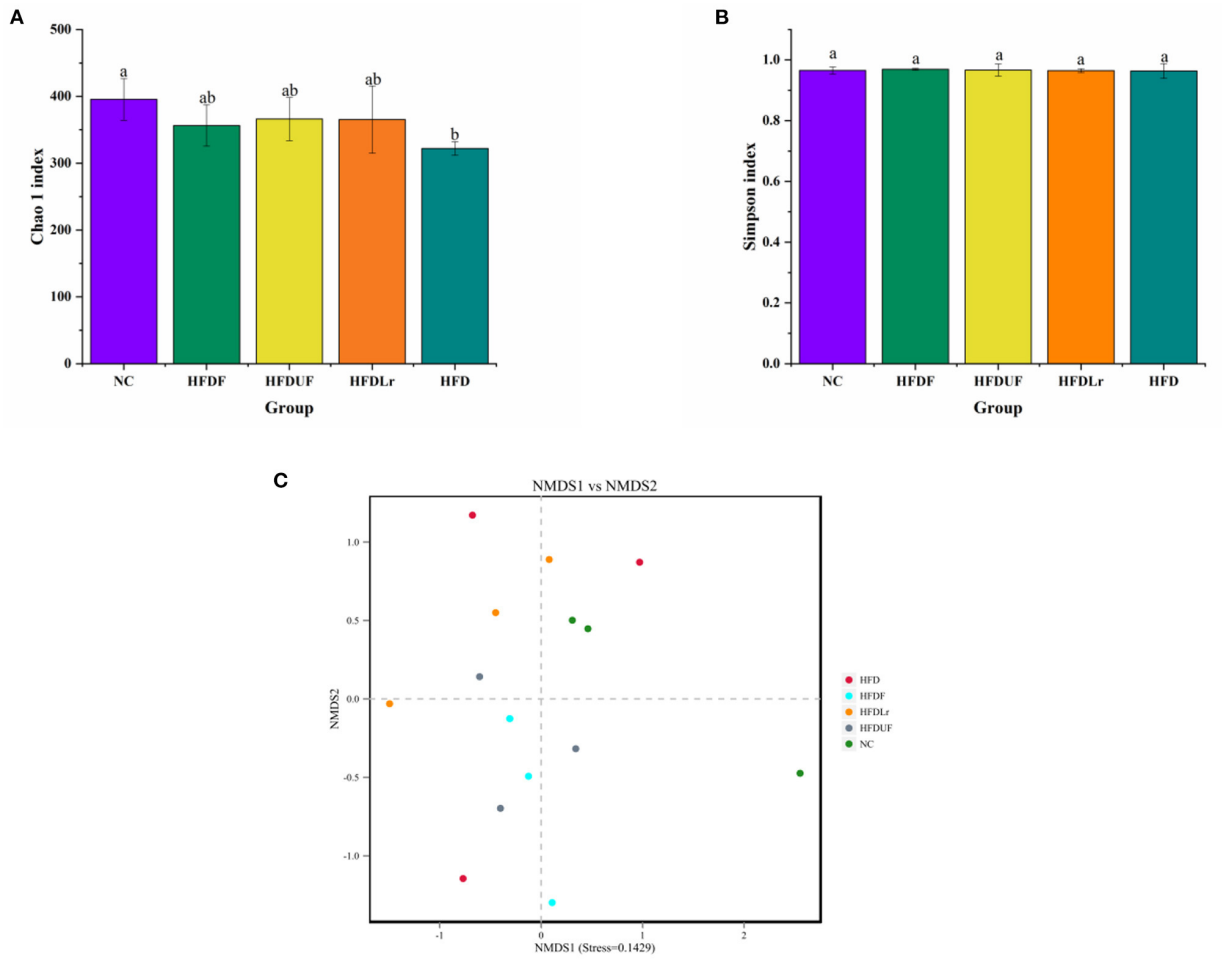
The effects of CSM on the diversity of intestinal microbiota in the HFD-fed mice. **(A)** Chao 1 index and **(B)** Simpson index to determine the alpha diversity of each sample group. **(C)** Non-metric multidimensional scaling (NMDS) for beta diversity analysis of each sample group. The superscript letters indicate significant differences between groups.

High-throughput sequencing of the V1-V9 region of the bacterial 16S rRNA gene was performed to evaluate the effects of different dietary interventions on the composition of intestinal microbiota. The rationality of the sequencing data was evaluated using a rarefaction curve, which was used to visualize species abundance and distribution. As shown in Figure 6, all of the curves gradually flattened as the sequencing number increased, indicating that the sample sequence was sufficient for data analysis. The Chao 1 and Simpson indices were used to estimate the species diversity and richness. As shown in Figure 7A, the Chao 1 index of the HFD group was significantly lower compared to the NC group (*p* < 0.05), indicating that the HFD reduced the abundance of intestinal bacteria in mice. However, there were no significant differences in the Chao 1 index between the mice in the HFDF, HFDUF, and HFDLr groups (*p* > 0.05). In addition, while there was no significant difference in the Simpson index among all treatment groups (*p* > 0.05), the Simpson index of the HFDF group was slightly higher than the other groups, indicating that CSM increased the diversity of intestinal microbiota in the mice (Figure 7B).

**Figure 8:**
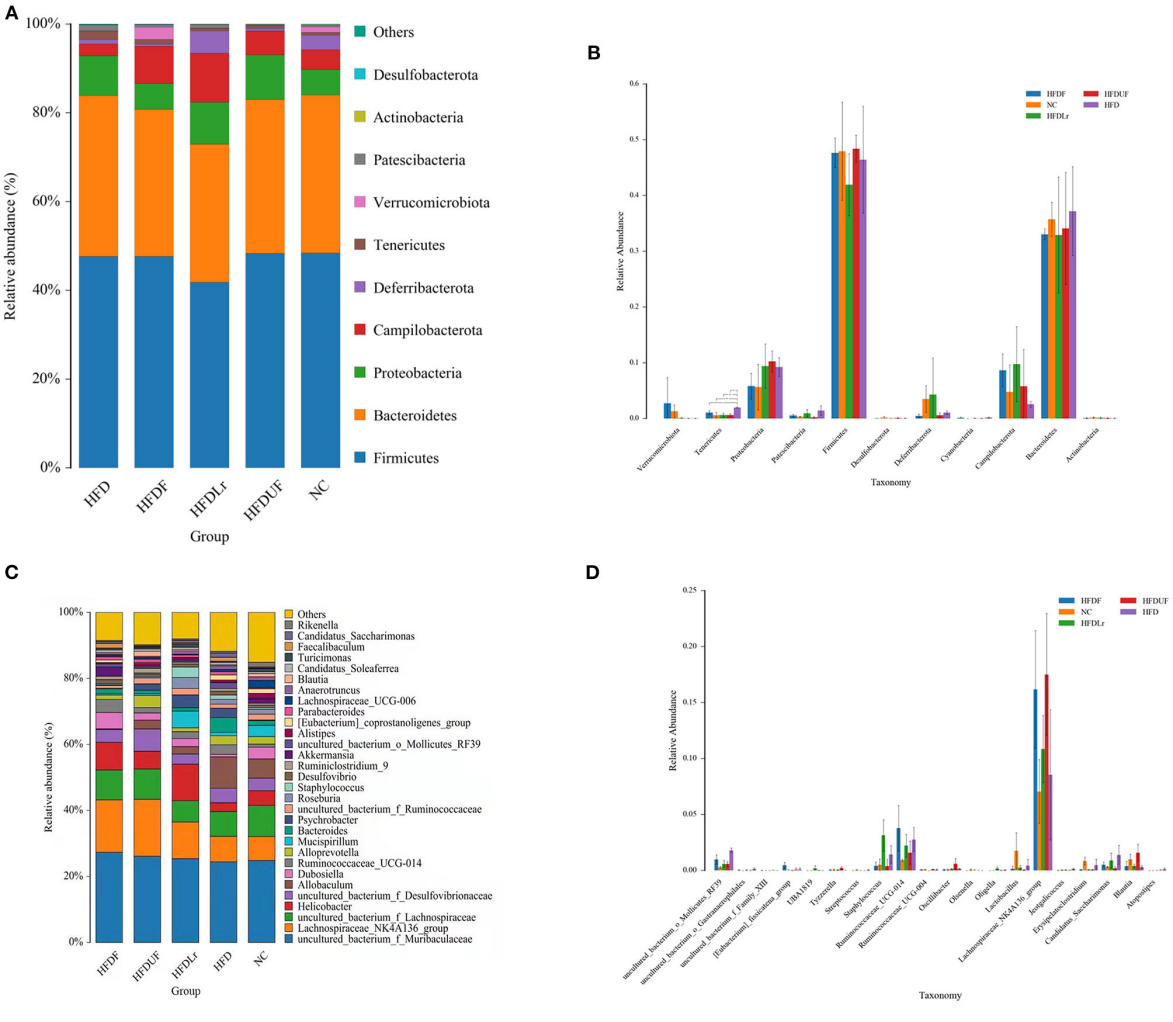
The effects of CSM on intestinal microbiota in the HFD-fed mice. **(A)** Relative abundance of the intestinal microbiota at the phylum level; **(B)** histogram derived from the ANOVA statistical analysis of the different treatment groups at the phylum level; **(C)** relative abundance of the intestinal microbiota at the genus level; and **(D)** histogram derived from the ANOVA statistical analysis of the different treatment groups at the genus level. *p < 0.05 indicates a significant difference between groups.

The structures of the gut microbiota among the mice in the various treatment groups were analyzed using Non-Metric Multidimensional Scaling (NMDS). NMDS analysis demonstrated a significant separation between the HFD and NC groups (Figure 7C), suggesting that HFD might alter the structure of intestinal microflora in mice. However, no significant separation appeared between the HFDF and HFDUF groups, while there was separated to some extent between the HFD group and the NC groups. More specifically, the relative abundance of dominant microorganisms at different classification levels were analyzed to assess specific changes in the intestinal microbiota as a result of CSM supplementation. At the phylum level (Figure 8A), the top 10 bacterial phyla in the gut of the mice were determined to be *Firmicutes, Bacteroidetes, Proteobacteria, Campilobacterota, Deferribacterota, Tenericutes, Verrucomicrobiota, Patescibacteria, Actinobacteria*, and *Desulfobacterota*. Bacterial taxonomic profiling indicated that the relative abundance of *Tenericutes* was significantly lower in the experimental treatment groups compared to the HFD group (*p* < 0.05). In addition, the levels of *Proteobacteria* and *Patescibacteria* in the treatment groups were also lower compared to the HFD group (Figure 8B). However, there were no significant differences in the abundance of *Firmicutes* and *Bacteroidetes* among all the groups (*p* > 0.05).

At the genus level, *Muribaculaceae, Lachnospiraceae, Helicobacter, Allobaculum, Dubosiella, Ruminococcaceae, Alloprevotella*, and *Mucispirillum* were the predominant gut microbes in the mice. In contrast to the other groups, the relative abundance of *Muribaculaceae, Lachnospiraceae_NK4A136_group, Lachnospiraceae* and *Dubosiella* were higher in the HFDF group (Figure 8C). Moreover, while the relative abundance of *Akkermansia* bacteria was significantly higher in the HFDF group (*p* < 0.05) compared to the other treatment groups, *Akkermansia* were barely detected in the mice in the HFDUF, HFDLr, and HFD groups, suggesting that not only did the HFD reduce the abundance of *Akkermansia* but also that the supplementation of the HFD with CSM could reverse this phenomenon. In the HFDUF group, *Desulfovibrionaceae* and *Alloprevotella* were found to be higher than in the other groups, and *Helicobacter* and *Mucispirillum* were the most abundant genera of microbes in the HFDLr group. Lastly, the abundance of *Lactobacillus* was lower in the gut of the HFD-fed mice compared to the NC group (Figure 8D).

### Correlation Between Intestinal Microbiota and Lipid Metabolism Parameters

**Figure 9:**
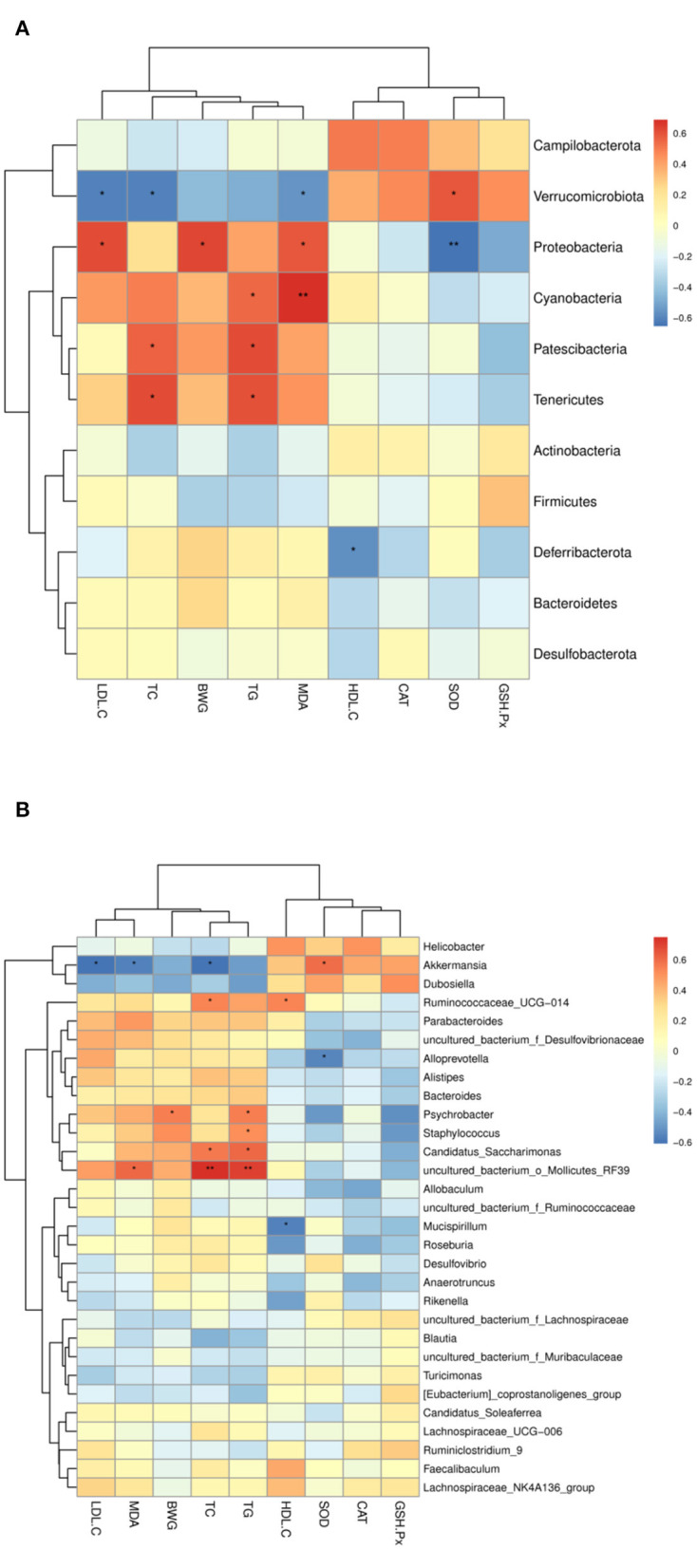
Heatmap of Spearman's correlation between the intestinal microbiota of significant differences and lipid metabolic parameters. **(A)** At the phylum level, **(B)** at the genus level. The intensity of the color representsthe degree of association between the gut microbiota and lipid metabolic parameters (blue represents a negative correlation, whereas red shows a positive correlation). *, represent significant differences (*p* < 0.05); **, represent extremely significant differences (*p* < 0.01).

To further clarify the possible relationship between lipid level parameters and intestinal microbiota, the Sperman's correlations were determined. As shown in Figure 9. At the phylum level (Figure 9A), *verrucomicrobiota* was positively correlated with SOD, and negatively correlated with TC, TG, and LDL-C; *Proteobacteria* was negatively correlated with SOD, and positively correlated with MDA, body weight gain (BWG), and LDL-C; *Patescibacteria* and *Tenericutes* were positively correlated with TG, TC; *Deferribacterota* was negatively correlated with HDL-C and positively correlated with BWG, TC and TG. At the genus level (Figure 9B), *Akkermansia* was positively correlated with SOD, but negatively correlated with TC, BWG, LDL-C and MDA. *Mucispirillum* was negatively correlated with HDL-C. *Uncultured_bacterium_o_Mollicutes_RF39* was positively correlated with TC, TG and MDA. Staphylococcus was positively correlated with TG. *Candidatus_Saccharimonas* was positively correlated with TG and TC. *Psychrobacter* is positively correlated with TG and BWG; *Ruminococcaceae_ucg-014* was positively correlated with HDL-C and TC.

## Discussion

Long-term consumption of HFD has the potential to alter lipid metabolism and induce hyperlipidemia, which can lead to an increase in the risk of other chronic diseases. In recent years, dietary intervention has been considered a noteable strategy for preventing and treating hyperlipidemia. In the present study, the effects of CSM on lipid metabolism and intestinal microbiota in HFD-fed mice were investigated. Supplementation of the HFD with CSM was found to effectively prevent weight gain and decrease the liver index in mice (Figure 1), suggesting that CSM might be efficacious in preventing and reversing obesity. This was corroborated by other studies, which also showed that other fermented products demonstrated anti-obesity properties (29, 30). In addition, unfermented coix seed milk and *L. reuteri* were also observed to alleviate weight gain in mice caused by a high-fat diet. The anti-obesity effect of coix seed had been confirmed in previous studies (31). Several studies on the anti-obesity of *L. reuteri* indicated that *L. reuteri* had both positive and negative regulation on the development of obesity (18, 32). Here, *L. reuteri* negatively regulates weight gain induced by a high-fat diet. Therefore, consumption of CSM does not raise consumer concerns about the development of obesity.

High serum levels of TC, TG, and LDL-C, as well as low serum levels of HDL-C, are often considered hallmark characteristics of hyperlipidemia. In this study, CSM significantly reduced the levels of TC, TG, and LDL-C and increased the levels of HDL-C in the serum of HFD-fed mice. These results were also confirmed by Wang et al. (25), who found that coix seed milk fermented with LAB reduced the levels of TC and LDL-C in the blood serum of hamsters. LDL-C and HDL-C are important carriers for solubilizing and transporting cholesterol through the body for lipid metabolism. LDL-C transports the cholesterol synthesized by the liver throughout the body *via* the blood. However, LDL-C is easily oxidized into the LDL, which adheres to the arterial wall, increasing the risk of atherosclerosis (33). On the contrary, HDL-C transports excess cholesterol from the blood to the liver for metabolism and can prevent LDL-C oxidation (34). In addition, the *L. reuteri* that was used to ferment the CSM might contribute to the regulation of the TC and TG levels. Taranto et al. also indicated that *L. reuteri* effectively prevented hypercholesterolemia in mice and increased the ratio of HDL to LDL (35). Thus, in this work, the regulation effect of CSM on serum lipid level was better than that of unfermented coix seed milk and *L. reuteri*. The possible explanation was that coix seed and *L. reuteri* played a synergistic effect *in vivo* and contributed to the regulation of serum lipid level.

It is well-known that the liver is the major organ for lipid and energy metabolism (36). Consumption of HFD can lead to the excessive accumulation of lipids in the liver, thereby causing damage that can hinder the function of the liver. According to the H&E staining and oil red O staining results of the mouse liver tissue (Figure 4), CSM significantly reduced the accumulation of lipids in the liver compared with the HFD group, indicating that CSM could be efficiacious in preventing the formation of fatty lesions in the liver. In addition, hepatic steatosis generally leads to an overproduction of reactive oxygen species (ROS) (37), which is an significant cause of non-alcoholic fatty liver disease (38). Meanwhile, the lipid peroxides formed by the reaction between ROS and polyunsaturated fatty acids can damage cell membranes, enabling the release of aldehyde compounds, such as MDA, from the cells (39). However, intracellular antioxidant enzymes, including SOD, CAT, and GSH-PX, can convert highly reactive ${\text{O}}_{2}^{•}$ and H_2_O_2_ species into benign O_2_ and H_2_O to minimize oxidative damage caused by ROS (40). Therefore, it is great significance to reduce the formation of MDA and increase the activity of antioxidant enzymes in liver to reduce oxidative damage. As expected, CSM significantly reduced the formation of MDA and increased the activities of SOD, CAT, and GSH-Px (Figure 3), indicating that CSM demonstrated a noticeable protective effect against oxidative damage caused by HFD. Although unfermented coix seed milk and *L. reuteri* also increased the activities of some antioxidant enzymes (CAT, GSH-Px) in liver and reduced the formation of MDA, the effect of CSM was more obvious. These beneficial effects may be partly attributed to some active components in CSM after fermentation, and further studies are needed to confirm this hypothesis.

Lipid and cholesterol metabolism in the liver are both regulated by enzymes and transcription factors (41). One previous study showed that HFD enhanced the expression of lipid biosynthesis-related genes in the liver (42). To determine the putative mechanism of the regulation of lipid metabolism by CSM on in HFD mice, we measured the changes in the mRNA expression of certain lipid metabolism-related genes, including ACC, FAS, HMGCR, and CYP7A1. As expected, the expression of ACC, FAS, and HMGCR mRNA in the mouse livers was significantly enhanced by the HFD. However, supplementation of the HFD with CSM led to a decrease in the expression of ACC, FAS, and HMGCR and an increase in the expression of CYP7A1 mRNA in the treatment group (Figure 5). ACC and FAS are key enzymes in lipid biosynthesis; ACC, which features two isoforms (ACC1 and ACC2), is involved in the synthesis and oxidation of fatty acids (43). One study indicated that ACC is the rate-limiting enzyme of fatty acid biosynthesis and is regulated by AMP-activated protein kinase (AMPK); phosphorylation of AMPK can prevent ACC dimerization and reduce the activity of ACC, thereby inhibiting the biosynthesis of fatty acids (44). The results of this study showed that CSM significantly reduced the expression of ACC in liver of HFD-fed mice. Therefore, we speculated that CSM might block ACC activity by increasing the activity of AMPK, thereby blocking the *de novo* production of fat and increasing the oxidation of fatty acids to regulate TG levels. FAS is also a rate-limiting enzyme of fatty acid biosynthesis, and the main function is to catalyze the synthesis of palmitate (16:0), which is an important precursor to triglycerides (45). Therefore, the downregulation of the expression of FAS might contribute to reducing the concentrations of TG. Recent research has shown that adlay seed oil inhibited the activity of FAS (46), which might have been a reason for the downregulation of FAS in the HFDF group. Additionally, cholesterol metabolism affects blood lipid levels. In the liver, cholesterol metabolism is regulated by the enzymes HMGCR and CYP7A1. HMGCR is the rate-limiting enzyme of cholesterol synthesis and is activated by SREBP-2 (2). Liu et al. (47) showed that the expression of SREBP-2 and HMGCR were increased in the liver of HFD-fed mice. In our study, the expression of HMGCR in the liver of the HFD mice group was also found to be higher than in the other groups, but this expression was significantly reduced after CSM intervention, suggesting that CSM downregulated the *de novo* biosynthesis of cholesterol by reducing the expression of HMGCR. In contrast, an upregulation of CYP7A1 expression will accelerate the conversion of cholesterol to bile acids and reduce the levels of TC (48). Our results showed that CYP7A1 expression was upregulated in the HFDF group compared with the HFD group, suggesting that CSM might promote the transformation of cholesterol to bile acid. However, the above conclusions were based on the results of mRNA levels, which needed to be confirmed by further experimental studies.

The intestinal microbiota plays a vital role in regulating the host's energy metabolism and material conversion, and is considered to be an important regulator of host health (47, 49). Accumulating evidence has indicated that the composition of the intestinal microbiota significantly influences lipid metabolism and may be a potential target for the treatment of dyslipidemia (21). Previous research has indicated that deleterious changes to the ratio of *Firmicutes* to *Bacteroides* in the intestines promoted abnormal lipid metabolism (12). Notably, Raman et al. found that there were no significant changes in the proportion of *Bacteroides* and *Firmicutes* phyla in the intestinal microbiota of obese NAFLD patients compared to the control group, but there were significant differences in the composition of certain families and genera of bacteria between these two groups (50). Wu et al. also suggested that changes in the composition of intestinal microbiota at the genus and species level might be more beneficial to host health compared to higher levels of classification (51).

In the present study, dietary intervention by CSM did not cause significant changes in the abundance of the Firmicutes and Bacteroides phyla of bacteria. However, CSM did induce changes in the composition of intestinal microbiota at the genus level in the HFD-fed mice (Figure 7). The bacterial taxonomy results showed that CSM intervention increased the abundance of *Muribaculaceae, Lachnospiraceae_NK4A136_group, Lachnospiraceae, Dubosiella* and *Akkermansia*. Spearman correlation analysis showed that these microflora were closely related to the improvement of lipid metabolism parameters (Figure 9). A previous study showed that the enzymes produced by *Muribaculaceae* family of bacteria could degrade carbohydrates and promote the production of short-chain fatty acids (SCFAs) (52). SCFAs are considered to be an important class of metabolites produced by the intestinal microbiota to regulate host physiology because they exhibit anti-inflammatory properties and help to balance the composition of glycolipids (53). In this work, although three dietary supplements (CSM, unfermented coix seed milk and *L. reuteri*) increased the relative abundance of *Muribaculaceae*, the relative abundance of *Muribaculaceae* was highest after CSM intervention. The results of spearman correlation analysis also showed that *Muribaculaceae* was positively correlated with GSH-Px and SOD, and negatively correlated with TC, TG and LDL-C. Therefore, promoting the production of SCFAs by intestinal flora may be one of the important ways that CSM regulates glycolipid balance in the body. Additionally, CSM intervention also increased the relative abundance of *Lachnospiraceae. Lachnospiraceae* is commonly found in the gut of mammals, especially humans, mice and cows (54). The *Lachnospiraceae* family includes known butyric acid producers (*Roseburia* and *Eubacterium*). Butyric acid is not only the main energy source of colonocytes, but also has the ability to promote the growth of *Lactobacillus* and *Bacteroidetes* in the colon (55). In our study, ingestion of CSM significantly increased the abundance of *Lachnospiraceae*, suggesting that CSM may facilitate the formation of butyric acid in the colon, thereby promoting the growth of beneficial microorganisms and maintaining the intestinal barrier. Furthermore, compared to HFD group, CSM significantly increased the relative abundance of *Dubosiella* (Figure 8C). It was reported that vitamin k_2_ supplementation could increase the abundance of intestinal *Dubosiella* and downregulate the relative expression of Atf4 in the thoracic aorta of mice, thereby inhibiting blood pressure (56). Ai et al. found that *Dubosiella* was significantly reduced in high-fat diet mice, and rice bran fermented with *Lactobacillus fermentum MF423* could significantly increase the abundance of *Dubosiella* and improve insulin resistance (57), they also found that *Dubosiella* was positively correlated with HDL, GSH-Px and SOD, and negatively correlated with TC and LDL. Similar results were also observed in our study, spearman correlation analysis indicated that *Dubosiella* was negatively correlated with TC, TG, LDL-C, BWG and MDA, and positively correlated with HDL-C, SOD and GSH-Px. Notably, in this study, the relative abundance of *Akkermansia* was significantly increased only in the intestine of the mice treated with CSM (Figure 8C). *Akkermansia* is a mucin-degrading genus of bacteria that exists in the mucosal layer of the intestines and can reverse metabolic disorders caused by HFD (58). Meanwhile, the protein AMUC-1100 present on the outer membrane of the *Akkermansia* muciniphila has been confirmed to enhance the effect of immune (59). Multiple studies have also shown that there was an inverse correlation between the abundance of *Akkermansia* bacteria and the indicidence of disease (60, 61). Consistent with these findings, we observed that *Akkermansia* was positively correlated with SOD, and negatively correlated with TC, BWG, LDL-C and MDA (Figure 9B). On the other hand, CSM supplementation reduced the abundance of *Desulfovibrionaceae*, which is a microorganism found to promote inflammation (62). In this study, *Desulfovibrionaceae* was positively correlated with LDL-C, MDA, BWG, TC and TG, while negatively correlated with SOD and CAT. Previous studies indicated that the relative abundance of *Ruminococca-ceae_UCG-014* was higher in obese rats, and positively correlated with obesity (63). Compared to HFD group, CSM significantly reduced the relative abundance of *Ruminococca-ceae_UCG-014* in this study (Figure 8C). Li et al. study also showed that curcumin intervention reduced the relative abundance of *Ruminococca-ceae_UCG-014* in the gut of high-fat diet mice (8). Our results were consistent with this report, and CSM showed the ability to reduce the relative abundance of *Ruminococca-ceae_UCG-014*. This study also found that CSM significantly reduced the abundance of *Psychrobacter* and *Staphylococcus*, which were positively correlated with TC and BWG. Previous studies showed that the abundance of *psychrobacter* and *staphylococcus* were increased in dyslipidemia mice (47, 64). The reduced abundance of *psychrobacter* and *staphylococcus* further confirms the ability of CSM to restore these gut microbiota. These results suggested that blood lipid levels were closely related to gut microbial composition. CSM may play a lipid-regulating role by affecting the gut microbial composition. However, the definite mechanisms need further research.

Nowadays, the main strategies for managing hyperlipidemia include drug therapy and dietary intervention. In the past and even today, drug therapy is still the preferred strategy for improving hyperlipidemia. However, the importance of dietary interventions in improving hyperlipidemia is still emphasized in addition to drug therapy. Valuable findings suggest that some dietary supplements (such as probiotics, functional foods) can improve hyperlipidemiar (9, 16, 24). Our results also confirmed the feasibility of probiotic-fermented foods for improving dyslipidemia induced by HFD. Consumption of these foods can regulate lipid metabolism and reverse gut dysbiosis induced by HFD. Meanwhile, some of the active compounds produced by fermentation may help improve blood lipid levels. Therefore, increasing the intake of these foods may help reduce the risk of hyperlipidemia.

In conclusion, CSM could prevent hepatic fat accumulation and improve blood lipid levels by regulating the expression of hepatic lipid metabolism-related genes in HFD-fed mice. In addition, CSM could regulate the composition of intestinal microbiota. Specially, CSM could increase the abundance of some beneficial bacteria and inhibit the growth of pathogenic bacteria, which would be one of important anti-hyperlipidemic mechanisms. We also demonstrated that the relative abundance of some microbiota were closely related to lipid parameters. Our results suggested that CSM might serve as a novel and promising dietary supplement that can be used to ameliorate hyperlipidemia and intestinal diseases caused by HFD. However, more in-depth and extensive clinical studies are needed to complement and validate these results.

## Data Availability Statement

The original contributions presented in the study are included in the article, the 16S sequencing data have been upload to NCBI SRA with accession number PRJNA858265, further inquiries can be directed to the corresponding author.

## Ethics Statement

The animal study was reviewed and approved by subcommittee of experimental animal ethics at Guizhou University.

## Author Contributions

ZY: data curation, formal analysis, investigation, methodology, visualization, and writing—original draft. XZ and JR: methodology. AW: writing—review and editing. LQ: conceptualization, writing—review and editing, funding acquisition, and supervision. YZ: conceptualization, funding acquisition, and supervision. All authors have read and agreed to the published version of the manuscript.

## Funding

The research was funded by the Agriculture Committee of Guizhou Province [grant No. (2018) 81].

## Conflict of Interest

The authors declare that the research was conducted in the absence of any commercial or financial relationships that could be construed as a potential conflict of interest.

## Publisher's Note

All claims expressed in this article are solely those of the authors and do not necessarily represent those of their affiliated organizations, or those of the publisher, the editors and the reviewers. Any product that may be evaluated in this article, or claim that may be made by its manufacturer, is not guaranteed or endorsed by the publisher.
